# Analysis of the efficacy and influencing factors of preoperative P‐SOX neoadjuvant chemotherapy regimen for progressive gastric cancer—construction of a clinical prediction model

**DOI:** 10.1002/cam4.5977

**Published:** 2023-04-25

**Authors:** Long Feng, Lei Shao, Shuangshuang Sun, Chengwu Zhang, Baojia Cai

**Affiliations:** ^1^ Department of Gastrointestinal Oncology Affiliated Hospital of Qinghai University Xining China; ^2^ Graduate School of Qinghai University Xining China

**Keywords:** efficacy, forecast, gastric cancer, influencing factors, neoadjuvant chemotherapy

## Abstract

Preoperative neoadjuvant chemotherapy is one of the most common treatments for patients with advanced gastric cancer that cannot be completely removed by surgery. Nab‐paclitaxel is a nano‐formulation of paclitaxel that has been shown to be effective in treating stomach cancer. In addition, oxaliplatin + S‐1 (SOX) has been a first‐line chemotherapy regimen for gastric cancer, and it has the effect of tumor downstaging, improving the R0 resection rate, and reducing the postoperative recurrence rate, but the side effects are significant. During the application of oxaliplatin, obvious gastrointestinal reactions such as nausea and vomiting can be observed. There may also be blood system side effects such as leukopenia and thrombocytopenia, as well as serious adverse reactions such as peripheral neuropathy. Therefore, we reduced the amount of oxaliplatin in SOX and added nab‐paclitaxel on the basis of this, in order to increase the efficacy while reducing the side effects of SOX regimen. We selected 192 patients with advanced gastric cancer admitted to the Department of Gastrointestinal Oncology of Qinghai University Hospital from July 2019 to February 2022, and all were treated with nab‐paclitaxel plus oxaliplatin + S‐1 neoadjuvant chemotherapy regimen, and underwent further surgery after chemotherapy. The tumor regression grade (TRG grade) and response evaluation criteria of solid tumor 1.1 (RECIST1.1) were taken as the dependent variables. According to TRG classification, 120 patients were effective (grade 0, 1, 2 = 62.50%, age: 55.63 ± 9.02 years), 72 patients were ineffective (grade 3 = 37.50%, 55.82 ± 9.21 years), and the effective rate of chemotherapy was 62.50%. According to RECIST1.1, 116 patients were effective (CR + PR = 60.42%, mean age 55.84 ± 9.02 years), 76 patients were ineffective (SD + PD = 39.58%, 55.47 ± 9.19 years), and the effective rate was 60.42%. The factors *p* < 0.2 in univariate logistic regression analysis were included in multivariate logistic regression analysis, and *p* < 0.05 was the statistical difference, and statistically significant factors were screened out for modeling and plotted the nomogram. Among them, in the tumor regression grade, the final factors related to effective chemotherapy are the degree of differentiation, cT. stage, tumor diameter, chemotherapy cycle, and the final factors related to effective chemotherapy in the solid tumor response evaluation criteria are the degree of differentiation, cT. stage, tumor diameter. Therefore, we conclude that the regimen of nab‐paclitaxel combined with oxaliplatin and S‐1 has certain positive significance in the treatment of advanced gastric cancer.

## Graphical abstract

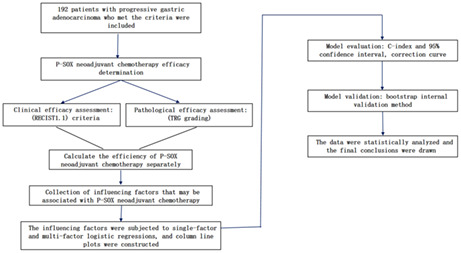

## 1 INTRODUCTION

In recent years, gastric cancer (GC) has become the fifth most common cancer all over the world, and its mortality rate is the fourth in the world.^1^ Risk factors for GC include H. pylori infection, family history of gastrointestinal cancer, consumption of preserved and smoked foods, smoking, and postgastrectomy surgery, but the pathogenesis has not been fully determined.^2^ Nowadays, despite some progress in the diagnosis and treatment of GC, patient prognosis has only minimally improved relative to the previous.^3^

For the treatment of GC, it has been shown that pre‐surgical neoadjuvant chemotherapy improves overall survival (OS) in patients with resectable GC compared to surgery alone.^4^ In fact, GC is often found by metastasis, and different routes of recurrence or metastasis usually mean different survival outcomes,^5^, ^6^ distant lymph nodes are a common site for advanced GC metastases.^7^ Moreover, the short survival time of GC with lymph node recurrence, similar to hematogenous recurrence,^6^ greatly reduces the success rate of radical surgical resection. Therefore, for most patients with advanced gastric cancer, prior neoadjuvant chemotherapy to regress the tumor before surgery is necessary.

At present, the first‐line neoadjuvant chemotherapy regimen for advanced gastric cancer is relatively mature, and commonly used drugs include oxaliplatin, 5‐FU, cisplatin, etc. It is undeniable that oxaliplatin + S‐1 (SOX) is the first‐line chemotherapeutic agent for the treatment of advanced gastric cancer in Asia.^8^ It has a good clinical response rate of 55%–59% in unresectable or recurrent gastric adenocarcinoma, and its use as a preoperative neoadjuvant chemotherapy regimen for GC has been proven to be reasonable.^9^ However, conventional chemotherapy drugs like these still have limited clinical use, not only because they do not achieve the desired therapeutic effect, but more importantly because of their significant systemic adverse reactions.^10^ Oxaliplatin, for example, has obvious curative effect on gastric cancer, colorectal cancer, breast cancer, and many other cancers, but the side effects (such as nausea, vomiting, leukopenia, peripheral neuropathy, etc.) of its regular dosage should not be ignored.^11^, ^12^ Another example is paclitaxel (PTX), although it is widely used in chemotherapy for GC, it can cause not only postchemotherapy myelosuppression and neurotoxicity, but also systemic hypersensitivity reactions,^13^ which can seriously affect the normal life of patients after chemotherapy. Therefore, in order to overcome the drug side effects, the nano‐formulation of albumin paclitaxel (nab‐paclitaxel), which has been studied in recent years, was introduced and used in clinical practice.^14^ Nab‐paclitaxel not only improves the objective remission rate of cancer after chemotherapy, but also shortens the injection time and reduces chemotherapy side effects.^15^ Moreover, there was a study reported the positive effects of using nab‐paclitaxel in combination with oxaliplatin in the treatment of advanced gastric cancer.^16^

Therefore, in this study, we took oxaliplatin reduction with significant side effects in SOX regimen and added nab‐paclitaxel on top of it, to predict the therapeutic effect of nab‐paclitaxel combined with oxaliplatin + S‐1 (P‐SOX) in patients with advanced gastric cancer and analyze the related influencing factors, and discuss and analyze. Construct nomogram to predict the therapeutic effect of nab‐paclitaxel in combination with oxaliplatin + S‐1 (P‐SOX) for patients with advanced gastric cancer. To explore whether the combination can be used in first‐line chemotherapy for advanced gastric cancer.

## 2 PATIENTS AND METHODS

### 2.1 Patients

Patients with advanced gastric cancer admitted to the Department of Gastrointestinal Oncology at the Affiliated Hospital of Qinghai University from July 2019 to February 2022 were included, and all patients received P‐SOX neoadjuvant chemotherapy regimen and were treated surgically. All patients met the following inclusion criteria: (1) Patients with primary gastric adenocarcinoma diagnosed as stage II‐III by imaging and endoscopic pathological tissue biopsy, without other malignant tumors in combination, without distant organ metastases (According to the 8th edition of the AJCC TNM Staging Criteria of the International Union Against Cancer (UICC)),^17^ and feasible for surgical R0 resection (no residual tumors under the naked eye or microscope); (2) Two to four cycles of preoperative neoadjuvant chemotherapy, all underwent surgical treatment in our hospital (According to the Clinical Practice Guidelines for Gastric Cancer CSCO of the Chinese Society of Clinical Oncology)^18^; (3) Primary tumor lesions with size measurable by CT and MRI and all underwent postoperative pathological biopsy. Exclusion criteria: (1) Patients who had received radiotherapy, neoadjuvant chemotherapy, biological therapy, or surgery for other malignancies; (2) Patients with incomplete or missing case data or imaging data that could not accurately measure the tumor diameter. A total of 192 eligible patients, including 160 males and 32 females, were included. Demographic characteristics (age and sex), overall status (American Society of Anesthesiologists score, body mass index), tumor characteristics (tumor location, diameter, degree of differentiation, TNM stage, depth of invasion, and lymph node metastasis), and neoadjuvant chemotherapy cycles were counted for these patients; efficacy determination: tumor regression grading (TRG grading: Ryan criteria),^19^ and response evaluation criteria in solid tumors (RECIST1.1).^20^ Written informed consent was obtained from all patients prior to enrollment. The trial was conducted in accordance with the Declaration of Helsinki. The protocol was approved by the institutional review boards of participating institutions.

### 2.2 Treatment

P‐SOX neoadjuvant chemotherapy regimen: Day 1 administration of albumin paclitaxel injection 135 mg/m^2^ intravenously for 3 h (slow drip at the beginning, pay attention to allergic reactions in the first 10 min); Day 1 oxaliplatin injection 85 mg/m^2^ intravenous drip 2–4 h; Day 1–14 oral S‐1, according to the body surface area BSA <1.25 m^2^, 40 mg/time, bid; BSA 1.25–1.5 m^2^, 50 mg/time, bid; BSA > 1.5 m^2^, 60 mg/time, bid; half an hour after breakfast and dinner orally, 21 days for one cycle. Depending on the patient's tumor characteristics, chemotherapy tolerance, and patient's willingness, neoadjuvant chemotherapy is administered for approximately two to four cycles. Upper gastrointestinal endoscopy and CT were performed after neoadjuvant chemotherapy to assess the resectability of the tumor. If the tumor is resectable, radical gastrectomy is performed 3–4 weeks after the last preoperative chemotherapy.

### 2.3 Clinical response and pathological assessment

Clinical efficacy assessment: The clinical efficacy of chemotherapy was evaluated by measuring the maximum tumor diameter (measured under the same gastric filling state) before (1 day before chemotherapy) and after (3 weeks after the final neoadjuvant chemotherapy) using CT and comparing the baseline tumor size with the postchemotherapy tumor size. The clinical efficacy of chemotherapy was evaluated according to the response evaluation criteria in solid tumors (RECIST1.1): complete response (CR) was defined as the disappearance of all target lesions with a short pathological lymph node diameter <10 mm; partial response (PR) was defined as a reduction of ≥30% compared with the total length of the baseline lesions; stable disease (SD) as a reduction of <30% or an increase of <20% compared to the total length of the lesion at baseline. Progressive disease (PD) was defined as an increase of ≥20% in the total length of the target lesion or the appearance of a new lesion. CR and PR were considered as effective groups, and SD and PD were considered as ineffective groups.

Pathological efficacy assessment: by postoperative pathological biopsy to see the extent of tumor necrosis or disappearance in relation to the estimated total amount of tumor, based on tumor regression grading (TRG grading: Ryan criteria): 0: no tumor residue (complete regression); 1: only a small amount of single cancer cell residue was seen; 2: tumor regression but with partial residue; 3: indicating no tumor regression; grading 0, 1, and 2 were identified as effective group, and grade 3 as ineffective group.

### 2.4 Statistical methods

Statistical analyses were performed using R software (version 4.12 http://www.r‐project.org/). The packages in R used in this study were all version 4.13 (rms package, rmda package, Hmisc package, caret package). Tumor regression grading (TRG grading) and response evaluation criteria in solid tumors1.1 (RECIST1.1) were used as dependent variables, respectively, and factors with *p* < 0.2 in the univariate logistic regression analysis were included in the multifactorial logistic regression analysis, and *p* < 0.05 was considered a statistically significant difference, and the statistically significant factors were screened out for modeling and plotted on nomogram. The prediction model was evaluated by calculating the C‐index and 95% confidence interval and plotting the calibration curve. 1000 replots with put‐back were performed on the original data set by bootstrap internal validation method to calculate the C‐index and 95% confidence interval.

## 3 RESULTS

### 3.1 Efficacy of neoadjuvant chemotherapy

One hundred and ninety‐two patients were enrolled and evaluated for efficacy. The patient characteristics were as follows: 160 males (83.3%) and 32 females (16.7%), including 53 stage IIa, 59 stage IIb, and 80 stage III patients; 120 patients were effective (grade 0, 1, 2 = 62.50%, age: 55.63 ± 9.02 years) and 72 patients were ineffective (grade 3 = 37.50%, 55.82 ± 9.21 years) as assessed by TRG classification. The chemotherapy efficiency was 62.50%, with 116 effective (CR + PR = 60.42%, age 55.84 ± 9.02 years) and 76 ineffective (SD + PD = 39.58%, 55.47 ± 9.19 years) cases in the RECIST1.1 assessment, the chemotherapy efficiency was 60.42% (Table 1).

**TABLE 1:** Clinical characteristics of patients included in the TRG and RECIST1.1 groups.

Factors	TRG		RECIST1.1	
	Grades 0, 1, 2 (*n* = 120)	Grades 3 (*n* = 72)	CR + PR (*n* = 116)	SD + PD (*n* = 76)
Age	55.63 ± 9.02	55.82 ± 9.21	55.84 ± 9.02	55.47 ± 9.19
Sex				
Female	25 (20.8)	7 (9.7)	22 (20.0)	10 (13.2)
Male	95 (79.2)	65 (90.3)	94 (80.0)	66 (86.8)
Tumor location				
Diffuse type	10 (8.3)	5 (6.9)	9 (7.8)	6 (7.9)
Lower	41 (34.2)	27 (37.5)	41 (35.3)	27 (35.5)
Middle	37 (30.8)	26 (36.1)	34 (29.3)	29 (38.2)
Upper	32 (26.7)	14 (19.5)	32 (27.6)	14 (18.4)
Differentiation (before NACT)				
Poorly	75 (62.5)	64 (88.9)	71 (61.2)	68 (89.5)
Moderate and well	45 (37.5)	8 (11.1)	45 (38.8)	8 (10.5)
cT stage				
2	17 (14.2)	4 (5.6)	17 (14.7)	4 (5.3)
3	83 (69.2)	40 (55.6)	84 (72.4)	39 (51.3)
4	20 (16.6)	28 (38.9)	15 (12.9)	33 (43.4)
cN stage				
Negative	25 (20.8)	17 (23.6)	27 (23.3)	15 (19.7)
Positive	95 (79.2)	55 (72.4)	89 (76.7)	61 (80.3)
cTNM				
III	45 (37.5)	35 (48.6)	40 (34.5)	40 (52.6)
IIa	37 (30.8)	16 (22.2)	40 (34.5)	13 (17.1)
IIb	38 (31.7)	21 (29.2)	36 (31.0)	23 (30.3)
BMI				
18.5–24	74 (61.7)	50 (69.4)	75 (64.7)	49 (64.5)
<18.5 or >24	46 (38.3)	22 (30.6)	41 (35.3)	27 (35.5)
Tumor diameter				
<5 cm	85 (70.8)	32 (44.4)	82 (70.7)	35 (46.1)
≥5 cm	35 (29.2)	40 (55.6)	34 (29.3)	41 (53.9)
Chemotherapy cycles				
2–3	67 (55.8)	50 (69.4)	68 (58.6)	49 (64.5)
4	53 (44.2)	22 (30.6)	48 (41.4)	27 (35.5)
ASA				
1 and 2	94 (78.3)	61 (84.7)	91 (78.4)	64 (84.2)
3	26 (21.7)	11 (15.3)	25 (21.6)	12 (15.8)

Abbreviations: ASA, American Society of Anesthesiologists Score; BMI, body mass index.

*Note*: Tumor location classified as upper 1/3, middle 1/3, lower 1/3, diffuse TNM staging according to the American Cancer Consortium (AJCC).

### 3.2 Factors influencing the effectiveness of neoadjuvant chemotherapy

After univariate logistic regression analysis, factors associated with chemotherapy effectiveness were screened (*p* < 0.2), which were sex, differentiation, cT. stage, tumor diameter, chemotherapy cycles, cTNM in the assessment of pathological efficacy, and in the assessment of clinical response efficacy were differentiation, cT. stage, tumor diameter, cTNM (Table 2). When these factors were included in the multifactorial logistic regression analysis, the factors that were ultimately associated with effective chemotherapy in TRG were differentiation (moderate and well vs. poorly, OR: 4.276, 95% CI: 1.851–10.974, *p* = 0.001), cT. stage (cT4 vs. cT2, OR: 0.135, 95% CI: 0.024–0.678, *p* = 0.018), tumor diameter (≥5 cm vs. <5 cm, OR: 0.342, 95% CI: 0.171–0.670, *p* = 0.002), chemotherapy cycles (four vs. two to three cycles, OR: 2.158, 95% CI: 1.084–4.419, *p* = 0.031), and the final factor associated with effective chemotherapy in RECIST was differentiation (moderate and well vs. poorly, OR: 4.850, 95% CI: 2.103–12.479, *p* < 0.001), cT. stage (cT4 vs. cT2, OR: 0.155, 95% CI: 0.028–0.758, *p* = 0.025), and tumor diameter (≥5 cm vs. <5 cm, OR: 0.412, 95% CI: 0.208–0.805, *p* = 0.010) (Table 3).

**TABLE 2:** Univariate logistic analysis of factors influencing the efficacy of neoadjuvant chemotherapy.

Factors	TRG			RECIST1.1		
	OR	95% CI	*p*	OR	95% CI	*p*
Age	0.998	0.966–1.030	0.885	1.005	0.973–1.037	0.781
Sex						
Female	Reference			Reference		
Male	0.409	0.156–0.957	0.051	0.647	0.277–1.425	0.293
Tumor location						
Diffuse type	Reference			Reference		
Lower	0.759	0.217–2.389	0.647	1.012	0.308–3.137	0.983
Middle	0.712	0.202–2.254	0.573	0.782	0.237–2.430	0.673
Upper	1.143	0.309–3.874	0.833	1.524	0.438–5.086	0.495
Differentiation (before NACT)						
Poorly	Reference			Reference		
Moderate and well	4.800	2.208–11.666	0.001	5.387	2.480–13.091	0.001
cT stage						
2	Reference			Reference		
3	0.488	0.134–1.422	0.223	0.507	0.139–1.477	0.248
4	0.168	0.043–0.533	0.005	0.107	0.027–0.345	<0.001
cN stage						
Negative	Reference			Reference		
Positive	1.175	0.576–2.354	0.652	0.811	0.391–1.632	0.562
cTNM						
III	Reference			Reference		
IIa	1.799	0.872–3.810	0.117	3.077	1.460–6.784	0.004
IIb	1.407	0.707–2.837	0.333	1.565	0.794–3.122	0.198
BMI						
18.5–24	Reference			Reference		
<18.5 or >24	1.413	0.764–2.660	0.276	0.992	0.543–1.826	0.979
Tumor diameter						
<5 cm	Reference			Reference		
≥5 cm	0.329	0.178–0.602	<0.001	0.354	0.192–0.643	0.001
Chemotherapy cycles						
2–3	Reference			Reference		
4	1.798	0.977–3.374	0.063	1.281	0.707–2.344	0.417
ASA						
1 and 2	Reference			Reference		
3	1.534	0.722–3.445	0.279	1.465	0.698–3.220	0.324

**TABLE 3:** Multivariable logistic analysis of factors influencing the efficacy of neoadjuvant chemotherapy.

Factors	TRG			RECIST1.1		
	OR	95% CI	*p*	OR	95% CI	*p*
Sex						
Female	Reference					—
Male	0.464	0.162–1.193	0.126			—
Differentiation (before NACT)						
Poorly	Reference			Reference		
Moderate and well	4.276	1.851–10.974	0.001	4.850	2.103–12.479	<0.001
cT stage						
2	Reference			Reference		
3	0.372	0.082–1.447	0.170	0.645	0.147–2.450	0.534
4	0.135	0.024–0.678	0.018	0.155	0.028–0.758	0.025
Tumor diameter						
<5 cm	Reference			Reference		
≥5 cm	0.342	0.171–0.670	0.002	0.412	0.208–0.805	0.010
Chemotherapy cycles						
2–3	Reference					—
4	2.158	1.084–4.419	0.031			—
cTNM						
III	Reference			Reference		
IIa	0.644	0.224–1.837	0.409	1.067	0.377–3.088	0.903
IIb	0.739	0.309–1.728	0.488	0.706	0.299–1.621	0.418

### 3.3 Establishment, evaluation, and validation of Nomogram

**FIGURE 1:**
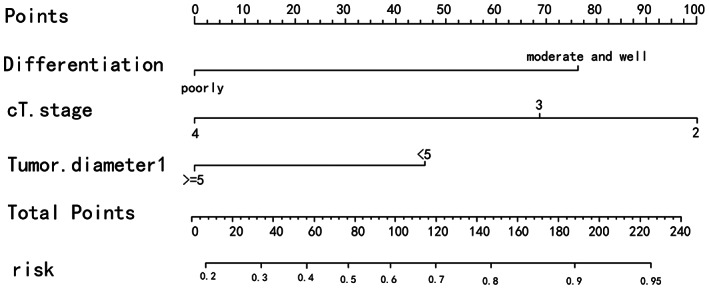
The nomination form model of RECIST1.1.

**FIGURE 2:**
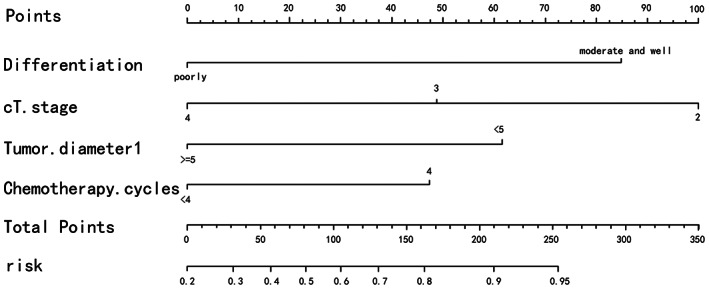
The nomination form model of TRG.

The nomogram to predict the efficacy of neoadjuvant chemotherapy will be established for the selected factors, ending with pathological efficacy evaluation and clinical efficacy evaluation, respectively. The sum of the corresponding scores of each factor will be marked by nomogram in the total score column and corresponding risk column. The greater the value, the greater the significance for the patient to receive neoadjuvant chemotherapy (Figures 1 and 2). Among them, patients with higher differentiation, more superficial invasion depth and tumor diameter <5 cm benefited best from neoadjuvant chemotherapy, while the relationship between chemotherapy cycles and chemotherapy efficacy, which showed an association in the TRG assessment, was not found in the RECIST1.1 assessment.

The performance of the nomogram model was validated for differentiation and calibration. The discrimination was evaluated by C‐index, which was 0.766 (95% CI, 0.699–0.833) in the nomogram built with pathological efficacy assessment as the outcome and 0.658 (95% CI, 0.592–0.724) in the bootstrap resampled 1000 times internal validation C‐index. The C‐index of the nomogram established with clinical efficacy assessment as an outcome was 0.773 (95% CI, 0.708–0.838) and the internal validation C‐index of bootstrap resampling 1000 times was 0.715 (95% CI, 0.649–0.781).

**FIGURE 3:**
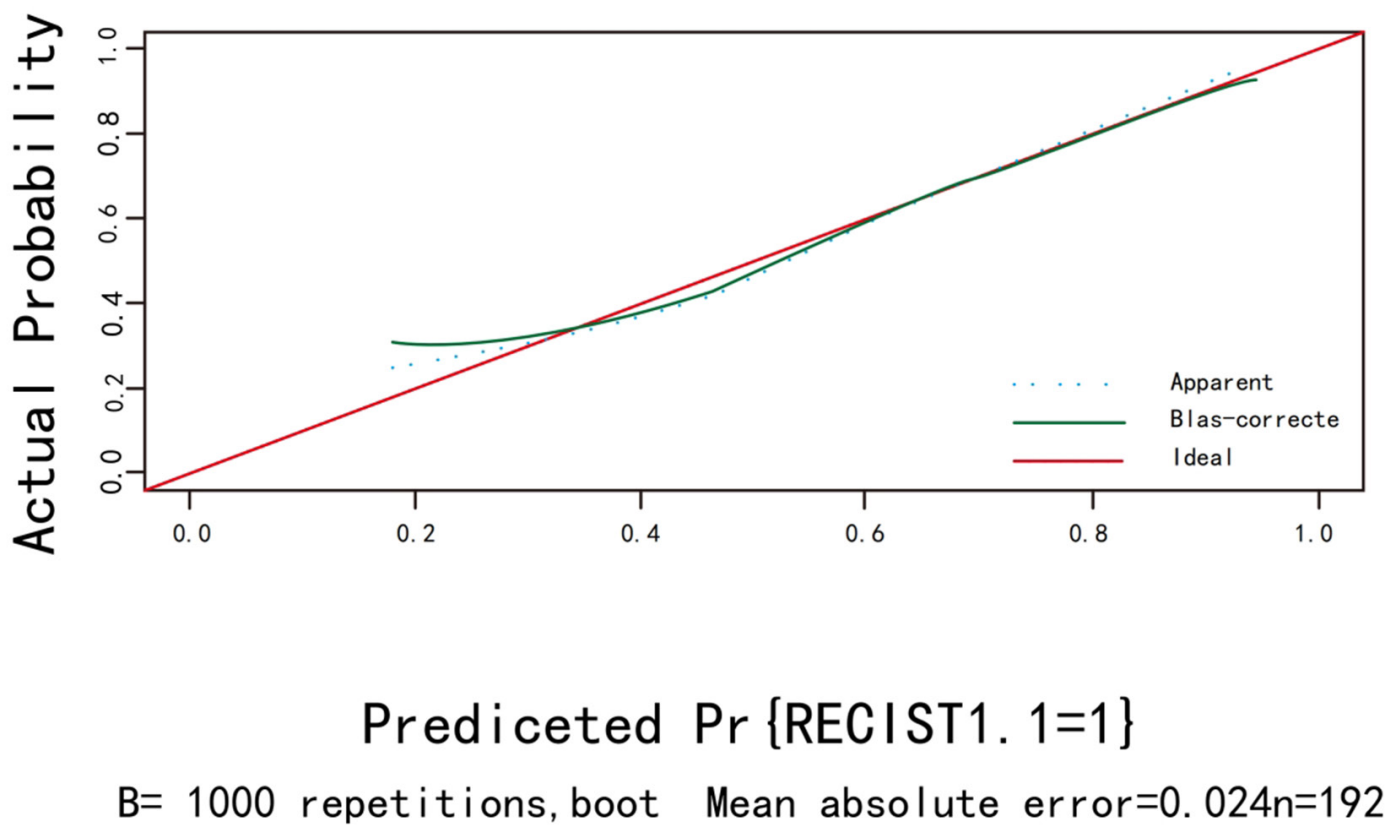
The calibration curve of RECIST1.1.

**FIGURE 4:**
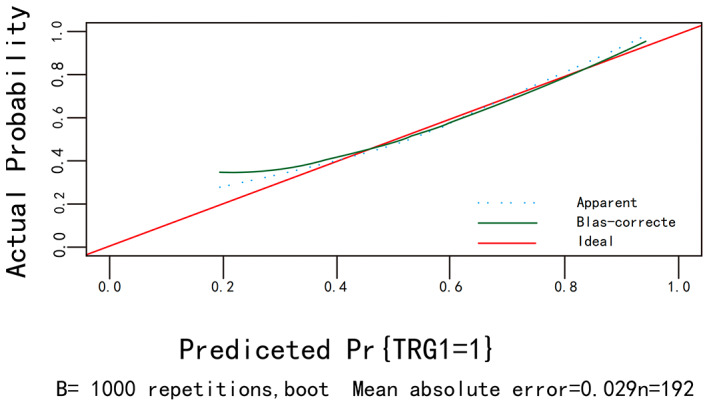
The calibration curve of TRG.

The calibration curves for bootstrap resampling 1000 times (Figures 3 and 4) showed good agreement between the predicted and observed results.

## 4 DISCUSSION

This study analyzed the efficacy of nab‐paclitaxel combined with oxaliplatin and S‐1 in patients with advanced gastric cancer and the factors influencing the efficacy through patient RECIST assessment and TRG assessment before and after chemotherapy. It is clearly pointed out in the clinical practice guidelines of CSCO for gastric cancer that the preoperative application of three‐cycle SOX chemotherapy regimen has positive significance for neoadjuvant therapy for gastric cancer.^18^ In this study, patients receiving two to four cycles of neoadjuvant chemotherapy were included according to the efficacy of patients and individual acceptance of chemotherapy. Through analysis, it is concluded that the efficiency of chemotherapy was 60.42% and 62.50% for RECIST1.1 and TRG, respectively. In a study on the efficacy of SOX in the treatment of gastric cancer, the effective rate of SOX was very close to 62.60%.^21^ Furthermore, it has been suggested that the addition of nab‐paclitaxel to the SOX regimen may enhance the antitumor effect on peritoneal metastases from advanced gastric cancer.^22^ Thus, we can conclude that the chemotherapeutic efficacy of P‐SOX regimen is not weaker than that of conventional SOX regimen, and it is superior to SOX regimen in some respects.

In the univariate and multifactorial logistic regression, we found that differentiation, cT. stage, and tumor diameter had a direct relationship with chemotherapy efficacy, but not with gender, age, ASA, BMI, tumor location, TNM stage, and whether lymph nodes were metastatic, but the relationship with chemotherapy cycles is still controversial. The individualized nomogram can provide a reference for physicians in the treatment process by quantifying the degree of influence factors associated with chemotherapy efficacy into specific scores and presenting them in the nomogram. Patients with higher degree of differentiation, shallow invasion depth, and tumor diameter <5 cm were considered to have the highest benefit in the efficacy of P‐SOX neoadjuvant chemotherapy, with the degree of differentiation and invasion depth having the greatest impact on chemotherapy efficacy. The high degree of differentiation and shallow depth of tumor infiltration undoubtedly indicate that the tumor has an early stage.^17^ Therefore, the results of our study indicate that patients with advanced gastric cancer with early stage benefit more from P‐SOX neoadjuvant chemotherapy. A Japanese report suggests that neoadjuvant chemotherapy with S‐1 is effective for stage II gastric cancer, while adjuvant chemotherapy with dual regimen is preferred for stage III gastric cancer.^23^ A study by David et al.^24^ also showed that neoadjuvant chemotherapy significantly reduced tumor size and depth of invasion and significantly improved progression‐free and overall survival for patients with early stage resectable gastric adenocarcinoma. Similarly, it is interesting to note that a randomized controlled study by Yoshiaki Iwasaki et al.^25^ showed that pre‐operative neoadjuvant chemotherapy using S‐1 plus cisplatin failed to demonstrate significant efficacy for stage IV or large stage III gastric cancer. In conclusion, although different chemotherapy regimens were adopted in this study, it also proved that patients with advanced gastric cancer with shallow tumor invasion and the high degree of differentiation had significant curative effects during chemotherapy. According to previous studies, a C‐index >0.7 indicates that the established nomogram have good accuracy and acceptable discriminatory power.^26^ Given that the C‐index of 0.766 (95% CI, 0.699–0.833) for the training group in the assessment of efficacy with TRG as the outcome was less stable compared with the internally validated C‐index of 0.658 (95% CI, 0.592–0.724) <0.7, while the C‐index in the nomogram established with RECIST1.1 as the outcome was 0.773 (95% CI, 0.708–0.838) and the internal validation C‐index was 0.715 (95% CI, 0.649–0.781), the training group possessed better stability compared with the validation group. The calibration curves showed good agreement between the predicted and observed results, so the nomogram presented in this study with RECIST1.1 as the efficacy assessment were more accurate, and the predicted impact factors and quantitative scores of impact factors were more reliable.

Some studies have shown that the prognosis of gastric cancer after radical gastrectomy is related to the stage, and the most critical factors are the depth of tumor invasion and lymph node metastasis.^27^, ^28^, ^29^ The aim of perioperative chemotherapy is to reduce the risk of local disease recurrence and improve overall survival (OS) by downstaging (T stage and N stage), improving CR rates, and treating micrometastatic disease.^24^, ^30^ Several studies have shown that neoadjuvant chemotherapy can significantly improve OS and progression free survival (PFS) in patients with advanced gastric cancer.^24^, ^31^, ^32^, ^33^ Therefore, an excellent neoadjuvant chemotherapy regimen will significantly benefit patients with advanced gastric cancer, including short‐term neoadjuvant efficacy and long‐term survival. In this study, P‐SOX chemotherapy regimen showed good efficacy in the evaluation of neoadjuvant efficacy. According to the nomogram, the patients with higher degree of differentiation, shallower invasion depth, and tumor diameter <5 cm were considered to benefit the most from the efficacy of P‐SOX neoadjuvant chemotherapy. It is well known that nab‐paclitaxel, which is formed by the binding of albumin to paclitaxel, is an intravenous preparation that can deliver higher doses in a shorter infusion time, has the advantages of good anti‐tumor effect, easy administration, and low drug side effects. It has been proven to be effective and low toxic, and is approved for the treatment of several cancer types. These include metastatic breast cancer, non‐small cell lung cancer, pancreatic cancer and advanced gastric cancer.^34^, ^35^, ^36^ Recently, a study compared the efficacy and safety of P‐SOX, DOF, and SOX neoadjuvant chemotherapy regimens for advanced gastric cancer. Patients with clinical stage T3‐4NxM0 were enrolled, and all three groups received two to four cycles of neoadjuvant chemotherapy followed by standard D2 radical resection. The efficacy of neoadjuvant chemotherapy was evaluated according to RECIST1.1 standard and TRG. Adverse events after neoadjuvant chemotherapy were evaluated based on CTCAE 5.0. Compared with DOF group and SOX group, P‐SOX group had the highest total response rate (CR + PR) and disease control rate (SD + PD). In terms of safety, there was no significant difference in the incidence of hematological and non‐hematological adverse events among the three groups (*p* > 0.05), and most of the hematological and non‐hematological adverse events occurred below grade 3. The results showed that there was no significant difference in the incidence of adverse events among p‐SOX group, DOF group, and SOX group (*p* > 0.05). Therefore, P‐SOX regimen has excellent safety and efficacy, and is expected to become a first‐line neoadjuvant chemotherapy regimen for patients with gastric cancer.^37^ In conclusion, nab‐paclitaxel combined with oxaliplatin and S‐1 has a certain positive significance in the treatment of advanced gastric cancer, and is expected to become a new chemotherapy regimen for advanced gastric cancer.

Limitations of this study: (1) Due to the short clinical application time of the P‐SOX chemotherapy regimen studied in this study, there is a lack of further evaluation of the prognosis of the included patients. In the future, more clinical trials can be conducted to explore the correlation between P‐SOX and the prognosis of patients. (2) At present, there are few relevant studies on the safety and efficacy of P‐SOX, and more clinical scholars and large‐scale trials are still needed to evaluate the advantages and disadvantages of this chemotherapy regimen.

## AUTHOR CONTRIBUTIONS

**Long Feng:** Data curation (equal); writing – original draft (equal). **Lei Shao:** Data curation (equal); writing – original draft (equal). **Shuang Shaung Sun:** Investigation (equal); validation (equal). **Chengwu Zhang:** Conceptualization (equal); visualization (equal). **Baojia Cai:** Methodology (equal); project administration (equal); supervision (equal); writing – review and editing (equal).

## CONFLICT OF INTEREST STATEMENT

The authors declare that they have no competing interests.

## ETHICS STATEMENT

Approval of the research protocol by an Institutional Reviewer Board. The study was conducted according to the principles of Declaration of Helsinki. The ethics committee of the Affiliated Hospital of Qinghai University approved this study.

## INFORMED CONSENT

Written informed consent was obtained from all patients.

## REGISTRY AND THE REGISTRATION NO. OF THE STUDY

SL‐2022‐035.

## Data Availability

The dataset used and analyzed during the current study is available from the corresponding author upon reasonable request.
